# Standard Minimum Dietary Diversity Indicators for Women or Infants and Young Children Are Good Predictors of Adequate Micronutrient Intakes in 24–59-Month-Old Children and Their Nonpregnant Nonbreastfeeding Mothers in Rural Burkina Faso

**DOI:** 10.1093/jn/nxaa360

**Published:** 2020-12-16

**Authors:** Loty Diop, Elodie Becquey, Zuzanna Turowska, Lieven Huybregts, Marie T Ruel, Aulo Gelli

**Affiliations:** International Food Policy Research Institute (IFPRI), Washington, DC, USA; International Food Policy Research Institute (IFPRI), Washington, DC, USA; Independent consultant for IFPRI, Dakar, Senegal; International Food Policy Research Institute (IFPRI), Washington, DC, USA; International Food Policy Research Institute (IFPRI), Washington, DC, USA; International Food Policy Research Institute (IFPRI), Washington, DC, USA

**Keywords:** minimum dietary diversity, micronutrient adequacy, Burkina Faso, rural areas, 24-h recall, children under five, women

## Abstract

**Background:**

Simple proxy indicators are needed to assess and monitor micronutrient intake adequacy of vulnerable populations. Standard dichotomous indicators exist for nonpregnant women of reproductive age and 6–23-mo-old children in low-income countries, but not for 24–59-mo-old children or pregnant or breastfeeding women.

**Objectives:**

This study aimed to evaluate the performance of 2 standard food group scores (FGSs) and related dichotomous indicators to predict micronutrient adequacy of the diet of rural Burkinabe 24–59-mo-old children and women of reproductive age by physiological status.

**Methods:**

A 24-h recall survey was conducted at dry season among 1066 pairs of children and caregivers. Micronutrient adequacy was evaluated by the mean probability of adequacy (MPA) of intake over 11 micronutrients. Proxy indicators were FGS-10 [10 food groups based on the FAO/FHI360 minimum dietary diversity for women (MDD-W) guidelines] and related MDD-W (FGS-10 ≥5); and FGS-7 [7 groups based on the WHO infant and young child (IYC) feeding MDD guidelines] and related MDD-IYC (FGS-7 ≥4).

**Results:**

FGS-10 and FGS-7 were similar across children and women (∼3 groups). FGS-10 performed better than FGS-7 to predict MPA in children (Spearman rank correlation = 0.59 compared with 0.50) and women of all 3 physiological statuses (Spearman rank correlation = 0.53–0.55 compared with 0.42–0.52). MDD-W and MDD-IYC performed well in predicting MPA >0.75 in children and MPA >0.6 in nonpregnant nonbreastfeeding (NPNB) women, but a 4-group cutoff for FGS-10 allowed a better balance between sensitivity, specificity, and proportion of correct classification. MPA levels for pregnant and breastfeeding women were too low to assess best cutoff points.

**Conclusions:**

MDD-IYC or an adapted MDD-W (FGS-10 ≥4 instead of FGS-10 ≥5) can be extended to 24–59-mo-old children and NPNB women in similar-diet settings. The inadequacy of micronutrient intakes in pregnant and breastfeeding women warrants urgent action. Micronutrient adequacy predictors should be validated in populations where a higher proportion of these women do meet dietary requirements.

## Introduction

Human diets are key to human health and the sustainability of our planet, and must be improved globally (1). There is a need for indicators to assess and monitor diet quality, including in vulnerable populations such as children under 5 y of age and women of reproductive age in developing countries.

Assessing diet quality requires measuring adequacy of nutrients and energy intakes using dietary assessment tools such as quantitative food records, dietary history, 24-h dietary recalls, or semiquantitative FFQs. These quantitative methods are often characterized by a high respondent burden, require well-trained interviewers, data management experts, and context-specific food composition tables, and most of them also require context-specific measure-to-weight conversion tools. Their implementation at the population level is time- and resource-intensive, and/or capacity-demanding. There is, therefore, a need for simpler tools to measure diet quality. Dietary diversity indicators were designed in response to this need and to reflect the diversity of food intake, which is one of the critical dimensions of dietary quality, along with proportionality (adequate proportions of certain nutrients such as energy from fat or sugar) and moderation (modest amounts of animal-sourced foods, highly processed foods, and foods high in energy, sugar, saturated/*trans*-fat, and salt) (1, 2).

Food group scores (FGSs), which consist of counting the number of food groups consumed over a given period, are relatively straightforward to measure and are now widely used to assess and monitor progress on dietary diversity in low-income countries (3). Several studies have highlighted the positive association between FGSs and micronutrient adequacy (such as the mean probability of adequacy, MPA) (4), or micronutrient density (5, 6). These studies led to the development, validation, and recommendation for international use of 2 standard dichotomous indicators of diet diversity: the minimum dietary diversity (MDD) for infants and young children (IYC) aged 6–23 mo (MDD-IYC), which requires the consumption over the past 24 h of 4 of 7 standard food groups (7); and the MDD for nonpregnant women of reproductive age (MDD-W), which requires the consumption over the past 24 h of 5 of 10 standard food groups (8, 9). However, evidence gaps remain on the validity of using these indicators and their related FGSs for other age groups and for pregnant or breastfeeding women. In children aged >2 y, some studies, using different food grouping systems, suggest that FGSs are good proxies of the micronutrient adequacy of the diet in settings as diverse as urban and rural Philippines (24–71-mo-old nonbreastfeeding children), urban Mali (13–58-mo-old children), urban and rural South Africa (1–8-y-old children), or rural Zambia (4–8-y-old children) (10–13). However, there is currently no standard recommendation for a food group indicator(s) validated for its (their) performance in predicting micronutrient adequacy in children aged ≥2 y, or in pregnant women.

This study aimed to fill some of these gaps by providing new evidence on the performance in children aged 24–59 mo and their mothers [nonpregnant and nonbreastfeeding (NPNB), pregnant, or breastfeeding] of the 2 standard dichotomous indicators currently recommended in specific populations: the MDD-IYC for children aged 6–23 mo, and the MDD-W for nonpregnant women, using data from rural Burkina Faso. The specific goals were to validate the performance of these indicators at predicting MPA in this sample and to determine whether 1 standard food group indicator and 1 standard cutoff point could be used across these age groups and for NPNB, pregnant, and breastfeeding women.

## Methods

### Country context

Burkina Faso is a low-income food-deficit country located in West Africa (14). The 2017 national nutritional surveillance survey found that 21% of children aged <5 y were stunted (height-for-age *z*-score < −2), and 9% were wasted (weight-for-height *z*-score < −2), and highlighted particularly poor infant and young child feeding (IYCF) practices (15). This survey also showed that, in 2017, only 18% of the Burkinabe children and 20% of women of reproductive age met the minimum dietary diversity (defined as having consumed 4 of 7 food groups for children and having consumed 5 of 10 food groups for women of reproductive age).

### Data sources

This study draws on data collected as part of the SELEVER (Soutenir l'Exploitation familiale pour Lancer l’Élevage des Volailles et Valoriser l’Économie Rurale) impact evaluation, including 2 rounds of surveys (baseline and 6-mo follow-up) undertaken prior to the intervention roll-out. The detailed SELEVER trial protocol has been published elsewhere (16). Briefly, the SELEVER cluster randomized control trial is an ongoing 5-y study evaluating the impact of an integrated agriculture-nutrition package of interventions on the diets, health, and nutritional status of women and children in Burkina Faso. The SELEVER study population includes 120 rural communities across 3 regions of Burkina Faso: Boucle de Mouhoun, Centre-Ouest, and Haut-Bassins. These regions were prioritized for the SELEVER roll-out based on the potential for the poultry production sector to meet the demand from urban markets (17). During the preparation stages of the SELEVER trial, 60 communes were selected from a pool of 79 communes available for the scale-up of the intervention (**Supplemental Figure 1**). The primary outcomes of the SELEVER trial are: *1*) MPA of diets for women and children (2–4 y at baseline) measured through dietary assessment using the interactive 24-h recall multiple-pass method at baseline, and after 6, 30, 36, and 48 mo; *2*) IYCF practices for children aged 0–2 y measured using interviews with caregiver at baseline, and after 6, 30, 36, and 48 mo; and *3*) household poultry production (output and sales) measured using interviews with caregiver at baseline, and after 6, 30, 36, and 48 mo. The 2 rounds of data were collected to allow for measurement of preintervention seasonal variations across the primary outcomes of the trial, with surveys timed during the dry and rainy seasons. The first survey was conducted from March to June 2017 in the dry season just before the first rains and prior to the main planting period. The follow-up survey was undertaken from September to October 2017, during the preharvest rainy season period. Both survey rounds included data collection at child, caregiver, household, and village level. The main analyses in this article use data from the dry season; data from the rainy season were used to assess the robustness of results and are presented as supplemental tables.

### Study population

This study analyzes dietary intake data of children aged 24–59 mo at the time of the baseline survey and of their primary female caregiver. The sampling and household selection protocol has been described in detail elsewhere (16). It involved a 2-stage sampling to accommodate budget constraints and different sample size calculations depending on the outcome of interest. The sample for dietary outcomes was part of the second stage of randomization (Supplemental Figure 1). Briefly, prior to the first baseline survey, a household census was conducted to identify all households with children in the 24–59-mo age group, and all the large poultry-producing households (defined as owning a poultry flock of >20 chickens/fowls) in the community. Twelve households were then randomly selected in 90 of the 120 rural communities for the dietary survey interviews, stratifying by poultry flock size status. Within each sampled household, an index child was randomly selected among all children aged between 24 and 59 mo. The caregiver could be the biological mother of the index child or another woman taking care of the child in the household.

Data were collected from a sample of 1066 pairs of caregivers and children (PCCs) enrolled at the baseline survey (**Supplemental Figure 2**). Collected data included dwelling features, sociodemographic characteristics of household members, food security information using the household food insecurity access scale classification (18), and dietary intakes of the PCC.

The study received ethical approval from the Comité d'Ethique pour la Recherche en Santé (national ethic comitee, Ministry of health/ Ministry of research) in Burkina Faso and the International Food Policy Research Institute IRB (Institutional Review Board) in Washington, DC. All caregivers provided written informed consent for themselves and their child as well.

### Dietary assessment protocol

Dietary intake data were collected using an interactive 24-h recall method (19). Caregivers were asked to report everything they and their child had eaten the previous day. After compiling a list of all foods that were eaten, a questionnaire was filled out using computer-assisted personal interviewing with information for each individual food item, including quantities consumed, quantities of leftovers, and detailed recipes for composite dishes. To facilitate the quantification of portion sizes and to avoid sharing food from a common pot, enumerators distributed 2 bowls and 2 plates to the caregiver 2 d before the interview and advised them to serve themselves and their index child all meals using these bowls or plates until the 24-h recall would take place. During the recall, portion sizes were estimated using the distributed plates and bowls and other common household measures, water volume, images, or clay or wooden models, if it was not possible to directly weigh a food replicate.

During the follow-up survey, data were collected from the same PCC using the same 24-h recall method. To capture the intraindividual variation of intakes necessary to estimate usual intakes, a second 24-h recall was undertaken on a nonconsecutive day in ∼16% of the sample during both survey rounds.

### Data

Food quantities were collected in grams, volume, prices paid for purchase, or in terms of household measures units and later converted to grams using context-specific conversion lists. Several of the context-specific conversion factors and all price-related conversion factors were collected during market surveys conducted concurrently with the household surveys.

Food quantity values obtained were converted into nutrients, using either the FAO West African food composition table (FCT) (20) or another FCT adapted to Burkinabe foods (21), after adjustment for micronutrient retention factors for cooked foods (22) and edible portions.

All individuals were included in the analysis except for 44 women (4% of the sample) who reported fasting for Lent (which was not common in our population), and 1 woman who fasted for other reasons and consumed only 1 cup of coffee during the previous day (Supplemental Figure 2). As recommended (23), to avoid introducing an unknown bias, we included in the analysis the overreporters (16% in women, 3% in children) and underreporters (11% in women, 8% in children) according to the Goldberg cutoff method (24).

### Micronutrient adequacy assessment

The micronutrient adequacy of individuals was assessed for 11 micronutrients: vitamin A, vitamin C, thiamin, riboflavin, niacin, vitamin B-6, vitamin B-12, folate, calcium, zinc, and iron. Vitamin A was expressed in micrograms of retinol activity equivalents and other micronutrients in micrograms or milligrams. The probability of adequacy (PA) for each micronutrient of interest was assessed through the probability approach (25) using the estimated average requirements (EARs) and SDs for women aged <18 y, women aged 19–49 y (pregnant, breastfeeding, or NPNB), and children aged 24–59 mo, based on WHO/FAO recommendations (26). For iron, whose distribution is generally skewed, we used the Institute of Medicine approach to calculate the PAs (27), accounting for the low bioavailability of iron (assumed to be 5%) in our context. We used the recommendations issued by the International Zinc Nutrition Consultative Group to calculate the PA for zinc, considering the lowest level of bioavailability (28). We used Institute of Medicine recommendations to calculate the PA for calcium (29). For the remaining nutrients (niacin, thiamin, riboflavin, folate, and vitamins A, C, B-6, and B-12), a Box–Cox transformation was used to obtain symmetrical distributions and, then, using the intraindividual and interindividual variances obtained from the second recall on a nonconsecutive day conducted in 16% of the households, the best linear unbiased predictor of an individual's usual intake was calculated (30). Depending on the relative size of the intraindividual to the interindividual variability in intake for each nutrient, the best linear unbiased predictor shrinks the person-level mean intake toward the overall group mean. The PA was then estimated for each micronutrient as the probability that an individual's usual intake is above the actual requirement for that micronutrient, the latter being along the normal distribution of requirements, with known EAR and SDs (25). However, for vitamin B-12, because the majority of zero values in its distribution did not allow the calculation of usual intakes, the actual intakes were used to estimate the PA. For each individual micronutrient, the mean PA at population level corresponds to the prevalence of individuals in that population covering their dietary requirements for the micronutrient. An overall MPA was then calculated for each individual by averaging the PA values across the 11 micronutrients considered.

### Dietary diversity indicators (proxy indicators)

The 24-h dietary recall data were used to derive FGSs. Each individual food was classified into a food group. Two validated classifications commonly used in the literature were used to build 2 FGSs. The first FGS (named FGS-10) was based on the minimum dietary diversity for women (MDD-W) guidelines whose food groups are the following: *1*) grains, white roots and tubers, and plantains; *2*) pulses; *3*) nuts and seeds; *4*) dairy; *5*) flesh foods; *6*) eggs; *7*) dark-green leafy vegetables; *8*) vitamin A–rich fruits and vegetables; *9*) other vegetables; and *10*) other fruits (8). This classification was shown to provide an FGS that is an adequate proxy of the MPA of nonpregnant women (9). The second FGS (FGS-7) was based on the IYCF minimum dietary diversity (MDD-IYC) guidelines and had the following groups: *1*) grains, roots, and tubers; *2*) legumes and nuts; *3*) dairy products; *4*) flesh foods; *5*) eggs; *6*) vitamin A–rich fruits and vegetables; and *7*) other fruits and vegetables (7). Oils (except vitamin A–enriched oils and red palm oil, which were classified among vitamin A–rich fruits and vegetables), drinks, and condiments were not classified into food groups. Foods whose total consumption during the day was <10 g were considered condiments and not included in the food groupings (31).

### Statistical analyses

We applied an identical analytical strategy to the sample of children and the 3 subsamples of caregivers: *1*) pregnant women; *2*) breastfeeding women; and *3*) NPNB women. First, we assessed the association between the discrete FGS-10 or FGS-7 and the MPA. Given the skewed distribution of the MPA distribution for both women and children, associations between the MPA and the 2 FGSs were assessed using Spearman rank correlation coefficients, both with and without adjustment for energy intake (32).

Then, the second analysis aimed to assess the performance of the FGS indicators in identifying individuals with low or higher MPA. For this purpose, the area under the receiver operating characteristic curve (AUC) values was calculated for the 2 highest possible cutoffs for MPA (including >0.6;  >0.7; >0.75;  >0.8; or >0.9) with ≥50 individuals above the cutoff points. Finally, sensitivity, specificity, and percentage of correct classification were calculated to assess the performance of various FGS cutoffs to screen for “higher” MPA, defined as >0.75 for children and >0.6 for NPNB women. This definition of a “higher” MPA is based on the distribution of MPA in the population and the cutoffs selected in previous studies for young children and NPNB women (4, 9, 10, 13). Sensitivity was defined as the percentage of children (or women) who had an FGS greater than a prespecified cutoff (e.g., 4 groups) among children (or women) with an MPA greater than the prespecified desired level (e.g., 0.6). Specificity was defined as the percentage of children (or women) whose FGS was lower than the cutoff considered among children (or women) who did not reach the required MPA threshold. These analyses were not carried out for pregnant or breastfeeding women because there were not enough women reaching a MPA >0.6. Robustness analyses were also conducted using various levels to define adequate MPA.

Because of the wide range of PA contributing to the MPA, we assessed the association between FGS and the PA for each micronutrient. We also assessed the performance (sensitivity, specificity, and percentage of correct classification) of the optimal FGS cutoff, as determined by the MPA analysis, to predict adequate micronutrient intake, defined as individual micronutrient PA >0.8.

We also repeated the whole analysis using the lean season data from the same PCC to assess the robustness of the findings. In addition, using both rounds, we used linear mixed-effects regression models to confirm the overall performance of the 2 FGSs to predict MPA. The models included random effects at the village and individual (child or woman) level, and fixed effects for the season and for child sex and age, maternal breastfeeding status, child or woman energy intake, and woman's age. The interaction of FGSs with the season was also tested. The assumption of normality of residuals from those regressions was assessed using the Shapiro–Wilk test (33). Finally, other robustness analyses were conducted without considering a minimum threshold to count a group.

## Results

### Sample description

Half of the children were male, and their average age was 41 mo (**Table 1**). Most women interviewed were biological mothers (99%) and their average age was 31 y. Only 18% of women had any formal schooling, the majority were housewives, and less than one-third were involved in an income-generating activity. Seventeen percent of women were pregnant and 41% were breastfeeding at the time of the survey. Most households depended on farming and were headed by a male household member (mean age = 44 ± 13 y). Almost half of the household heads had an income-generating activity in addition to farming activities, and less than one-quarter had formal schooling. Nearly one-third of the households were food insecure at the time of the survey [according to the household food insecurity access scale classification (18)].

**TABLE 1:** Sample description^1^

Children, *n*	1066
Age, mo	41 ± 10
Male, %	50
Sick during the recall day, %	9.0
Still breastfed, %	6.2
Women (15–49 y), *n*	1008
Biological mother of child, %	99
Age, y	31 ± 6.6
Married, %	94
Never been to formal school, %	82
Income-generating activity, %	29
Sick during the recall day, %	2.5
Breastfeeding, %	41
Pregnancy, %	17
Pregnant women, *n*	173
First trimester, %	34
Second trimester, %	36
Third trimester, %	29
Households, *n*	1064^2^
Household head	
Age, y	44 ± 13
Male, %	97
Never been to formal school, %	77
Income-generating activity, %	45
Food insecurity, %	32
Yesterday was a market day in the village, %	20
Yesterday was a market day in the village, *n*	216
A household member went to market yesterday, %	73

1 Values are mean ± SD, percentages (%), or frequencies (*n*).

2 There were 2 household questionnaires missing.

### Diet diversity and MPA

The mean FGS-10 and FGS-7 were very similar for children and women, with both women (regardless of their physiological status) and children consuming on average 3 food groups during the previous day (**Table 2**). Higher MPA values were found in children, with a mean MPA of 0.58, whereas the highest values for women were in the NPNB ones, with a mean MPA of 0.35. Compared with the sample of NPNB women, pregnant and breastfeeding women had lower MPAs, suggesting that women were not adjusting their diets to meet the additional requirements of pregnancy and lactation. Considering the 11 individual micronutrients, the prevalence of adequate intake was <50% for vitamin A, calcium, and vitamin B-12 in children; and for all micronutrients except iron (52–64%) in pregnant and breastfeeding women, and except zinc (51–88%) in all women.

**TABLE 2:** Food group scores, energy intakes, prevalence of adequate intake of 11 micronutrients and MPA for children and women^1^

	Children, *n* = 1066	NPNB women, *n* = 432	Breastfeeding women, *n* = 403	Pregnant women, *n* = 173
FGS-10	3.4 ± 1.2 (1–8)	3.3 ± 1.2 (1–7)	3.3 ± 1.2 (1–8)	3.3 ± 1.0 (1–6)
FGS-7	3.1 ± 0.93 (1–6)	3.0 ± 0.93 (1–5)	3.0 ± 0.97 (1–6)	3.0 ± 0.91 (1–5)
Energy, kcal/d	1300 ± 596 (133–4285)	2064 ± 826 (230–5967)	2208 ± 862 (58–5612)	1930 ± 727 (403–4047)
Iron	0.69 ± 0.31 (0–1)	0.39 ± 0.35 (0–1)	0.64 ± 0.37 (0–1)	0.52 ± 0.48 (0–1)
Vitamin A	0.46 ± 0.47 (0–1)	0.37 ± 0.45 (0–1)	0.23 ± 0.37 (0–1)	0.36 ± 0.45 (0–1)
Zinc	0.97 ± 0.13 (0–1)	0.88 ± 0.25 (0–1)	0.87 ± 0.27 (0–1)	0.51 ± 0.40 (0–1)
Calcium	0.17 ± 0.29 (0–1)	0.07 ± 0.21 (0–1)	0.10 ± 0.25 (0–1)	0.08 ± 0.22 (0–1)
Vitamin B-6	0.88 ± 0.29 (0–1)	0.47 ± 0.45 (0–1)	0.19 ± 0.35 (0–1)	0.18 ± 0.27 (0–1)
Vitamin B-12	0.05 ± 0.21 (0–1)	0.02 ± 0.14 (0–1)	0.03 ± 0.16 (0–1)	0.03 ± 0.17 (0–1)
Vitamin C	0.69 ± 0.45 (0–1)	0.45 ± 0.48 (0–1)	0.33 ± 0.45 (0–1)	0.43 ± 0.48 (0–1)
Folate	0.59 ± 0.46 (0–1)	0.19 ± 0.36 (0–1)	0.10 ± 0.26 (0–1)	0.02 ± 0.13 (0–1)
Riboflavin	0.56 ± 0.46 (0–1)	0.27 ± 0.39 (0–1)	0.14 ± 0.31 (0–1)	0.11 ± 0.28 (0–1)
Niacin	0.60 ± 0.41 (0–1)	0.29 ± 0.38 (0–1)	0.25 ± 0.36	0.13 ± 0.27 (0–1)
Thiamin	0.75 ± 0.39 (0–1)	0.50 ± 0.44 (0–1)	0.27 ± 0.36 (0–1)	0.27 ± 0.39 (0–1)
MPA	0.58 ± 0.22 (0–1)	0.35 ± 0.23 (0–0.96)	0.29 ± 0.2 (0–0.91)	0.24 ± 0.19 (0–0.78)

1 Values are mean ± SD (range). FGS-10, food group score based on the minimum dietary diversity for women (MDD-W) guidelines; FGS-7, food group score based on the infant and young child feeding minimum dietary diversity (MDD-IYC) guidelines; MPA, mean probability of adequacy; NPNB, nonpregnant nonbreastfeeding.

### Association between diet diversity and MPA

**FIGURE 1:**
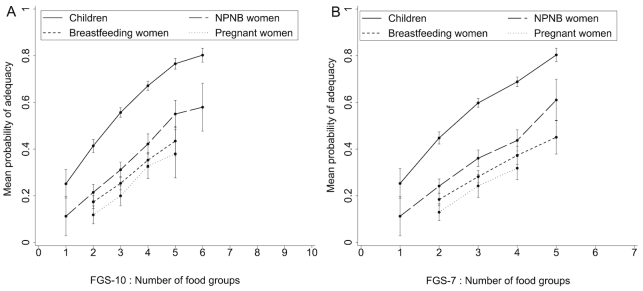
Mean probability of adequacy (MPA) by food group score in children and women: (A) FGS-10 and (B) FGS-7. Values are mean ± SEM. Data points representing <10 observations were excluded. Children, *n* = 1066; NPNB women, *n* = 432; breastfeeding women, *n* = 403; pregnant women, *n* = 173. FGS-7, food group score out of 7 food groups based on the WHO infant and young child feeding minimum dietary diversity (MDD-IYC) guidelines; FGS-10, food group score out of 10 food groups based on the FAO/FHI360 minimum dietary diversity for women (MDD-W) guidelines; NPNB, nonpregnant nonbreastfeeding.

Both FGS-10 and FGS-7 had a positive linear association with MPAs of children and women (**Figure 1**). Spearman correlation and partial correlation coefficients were all positive and statistically different from 0 (**Table 3**), though controlling for energy attenuated these associations. The correlation coefficients of FGS-10 were always higher than those of FGS-7, highlighting that in general FGS-10 was more strongly associated with MPA than FGS-7, both with and without controlling for energy intake (Table 3). There were positive and statistically significant associations between FGSs and PAs of the 11 micronutrients used to compute the MPA, although some coefficient values were rather low (range: 0.14–0.56).

**TABLE 3:** Correlation between FGS and the PAs of 11 micronutrients or MPA for children and women^1^

	Children (*n* = 1066)		NPNB women (*n* = 432)		Breastfeeding women (*n* = 403)		Pregnant women (*n* = 173)	
	FGS-10	FGS-7	FGS-10	FGS-7	FGS-10	FGS-7	FGS-10	FGS-7
MPA								
Without energy	0.59***	0.50***	0.53***	0.42***	0.54***	0.47***	0.55***	0.52***
With energy	0.40***	0.31***	0.31***	0.20***	0.38***	0.30***	0.39***	0.35***
PA iron	0.25***	0.25***	0.15***	0.14***	0.26***	0.28***	0.21***	0.26***
PA vitamin A	0.51***	0.38***	0.45***	0.33***	0.45***	0.35***	0.53***	0.42***
PA zinc	0.18***	0.17***	0.30***	0.27***	0.34***	0.34***	0.34***	0.37***
PA calcium	0.23***	0.20***	0.32***	0.28***	0.24***	0.24***	0.29***	0.27***
PA vitamin B-6	0.37***	0.32***	0.41***	0.33***	0.36***	0.33***	0.27***	0.23***
PA vitamin B-12	0.32***	0.38***	0.36***	0.43***	0.30***	0.35***	0.37***	0.41***
PA vitamin C	0.45***	0.35***	0.48***	0.34***	0.38***	0.28***	0.56***	0.43***
PA folate	0.47***	0.40***	0.45***	0.34***	0.27***	0.25***	0.24***	0.25***
PA riboflavin	0.38***	0.34***	0.23***	0.18***	0.27***	0.27***	0.29***	0.29***
PA niacin	0.50***	0.45***	0.46***	0.40***	0.51***	0.50***	0.46***	0.48***
PA thiamin	0.48***	0.40***	0.50***	0.38***	0.51***	0.47***	0.51***	0.46***

1 Values are Spearman rank correlation coefficients. Values unadjusted for energy intake are Spearman rank correlation coefficients. Values adjusted for energy intake are Spearman rank partial correlation coefficients (32). ***Significant compared with 0; *P* < 0.001. FGS-7, food group score based on the minimum dietary diversity for infant and young child feeding (MDD-IYC) guidelines; FGS-10, food group score based on the minimum dietary diversity for women (MDD-W) guidelines; MPA, mean probability of adequacy; NPNB, nonpregnant nonbreastfeeding; PA, probability of adequacy.

### Classification performance and preferred cutoffs

**FIGURE 2:**
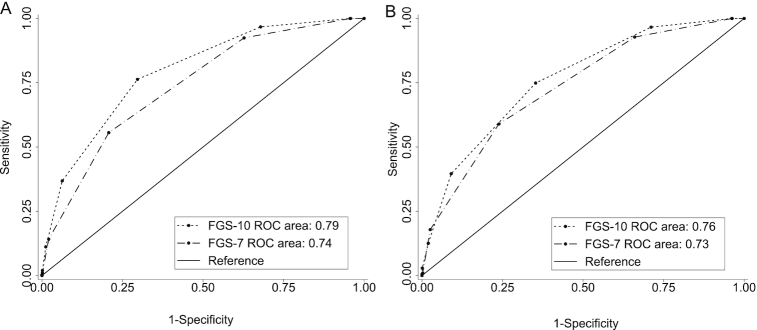
Receiver operating characteristic curves of FGS-10 (based on MDD-W) and FGS-7 (based on MDD-IYC): predictions for children of MPAs (A) >0.75 and (B) >0.8. FGS-7, food group score out of 7 food groups based on the WHO infant and young child feeding minimum dietary diversity (MDD-IYC) guidelines; FGS-10, food group score out of 10 food groups based on the FAO/FHI360 minimum dietary diversity for women (MDD-W) guidelines; MPA, mean probability of adequacy.

**FIGURE 3:**
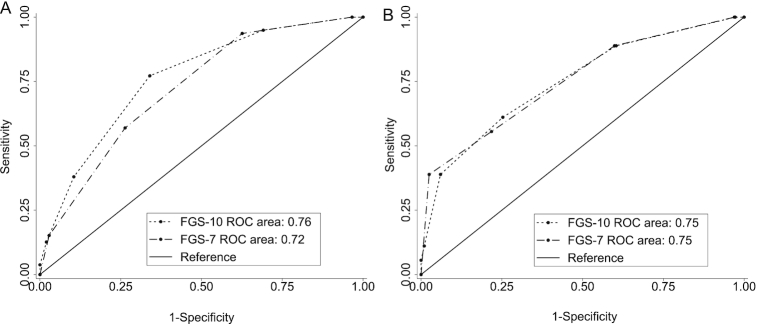
Receiver operating characteristic curves of FGS-10 (based on MDD-W) and FGS-7 (based on MDD-IYC): predictions for NPNB women of MPAs (A) >0.6 and (B) >0.7. FGS-7, food group score out of 7 food groups based on the WHO infant and young child feeding minimum dietary diversity (MDD-IYC) guidelines; FGS-10, food group score out of 10 food groups based on the FAO/FHI360 minimum dietary diversity for women (MDD-W) guidelines; MPA, mean probability of adequacy; NPNB, nonpregnant nonbreastfeeding.

To assess classification performances, AUCs were calculated for the 2 highest possible cutoffs for MPA with ≥50 individuals above the cutoff (**Table 4**). We considered the performance of various FGS cutoffs to screen for “higher” MPA, defined as >0.75 for children and >0.6 for NPNB women. In terms of accuracy, for both FGSs, AUCs >0.7 for children and NPNB women were found, suggestive of an acceptable predictive power for all possible food group cutoffs (**Figures 2** and **3**) (34). In general, AUCs for the FGS-10 were higher than or equal to those for the FGS-7.

**TABLE 4:** Percentages of children and women with MPA above selected cutoffs^1^

	Children (*n* = 1066)	NPNB women (*n* = 432)	Breastfeeding women (*n* = 403)	Pregnant women (*n* = 173)
MPA >0.5	66	26	13	12
MPA >0.6	53	18	7.7	4.1
MPA >0.7	37	11	4.5	1.7
MPA >0.75	29	7.9	2.5	1.2
MPA >0.8	19	4.2	2.0	0.0
MPA >0.9	3.8	0.69	0.5	0.0

1 Values are percentages. MPA, mean probability of adequacy; NPNB, nonpregnant nonbreastfeeding.

For the sample of children, MDD-W (i.e., FGS-10 ≥5) had an excellent specificity (94%), meaning that nearly all children with low MPA were identified as such by MDD-W (**Table 5**). However, several children with a high MPA did not meet the cutoff of 5 food groups, leading to poor sensitivity (37%). Applying a cutoff of ≥4 food groups to the FGS-10 optimized the sensitivity (∼76%), specificity (∼70%), and correct classification (∼72%). MDD-IYC (i.e., FGS-7 ≥4) was the best possible dichotomous indicator using FGS-7, with a good specificity (∼79%), a moderate sensitivity (∼56%), and 73% of children correctly classified.

**TABLE 5:** Summary of FGS characteristics relative to predicting MPA of children and NPNB women^1^

		Children, *n* = 1066, MPA >0.75				NPNB women, *n* = 432, MPA >0.6			
		*n*	Sensitivity	Specificity	Correctly classified	*n*	Sensitivity	Specificity	Correctly classified
FGS-10	(≥1)	1066	100	0.0	29	432	100	0.0	18
	(≥2)	1033	100	4.3	32	420	100	3.4	21
	(≥3)	811	97	32	51	319	95	31	43
	(≥4)	458	76	70	72	181	77	66	68
	MDD-W (≥5)	160	37	94	77	67	38	90	80
	(≥6)	43	11	99	74	17	13	98	82
	(≥7)	7	2.0	100	72	3	3.8	100	82
	(≥8)	1	0.3	100	72	0	0.0	100	82
	(>8)	0	0.0	100	71	—	—	—	—
FGS-7	(≥1)	1066	100	0.0	29	432	100	0.0	18
	(≥2)	1034	100	4.2	32	420	100	3.4	21
	(≥3)	759	92	37	53	295	94	37	48
	MDD-IYC (≥4)	327	56	79	73	138	57	74	71
	(≥5)	59	14	98	74	22	15	97	82
	(≥6)	4	1.0	100	72	0	0.0	100	82
	(>6)	0	0.0	100	71	—	—	—	—

1 Values of sensitivity, specificity, and correctly classified are percentages. FGS-7, food group score based on the minimum dietary diversity for infant and young child feeding (MDD-IYC) guidelines; FGS-10, food group score based on the minimum dietary diversity for women (MDD-W) guidelines; MPA, mean probability of adequacy; NPNB, nonpregnant nonbreastfeeding.

Because too few pregnant (7 of 173) or breastfeeding (31 of 403) women had an MPA >0.6, we could not meaningfully analyze the power of any indicator to predict higher MPA in these subsamples. Therefore, we limited our predictive power analyses on the capacity of FGSs in predicting higher MPA (>0.60) in NPNB women only (Table 5). MDD-W had an excellent specificity (90%) and high percentage of correct classification (80%), but a rather low sensitivity (38%). The cutoff of FGS-10 ≥4 food groups offered a slightly better balance between sensitivity (∼77%) and specificity (∼66%), and correct classification (∼68%). When using FGS-7, MDD-IYC performed well, with sensitivity at 57%, specificity at 74%, and correct classification at 71%.

Considering performances of FGSs to predict a PA >0.8 for each of the 11 micronutrients used to calculate the MPA, we noticed that MDD-W and MDD-IYC had a very good specificity, which is our main focus because we judged it is more important to make sure that individuals with low MPA are not classified as having an adequate diet (given the adverse consequences of micronutrient deficiencies on the health of individuals) (**Table 6**). As for the prediction of MPA, a cutoff of 4 groups allowed a better balance between sensitivity, specificity, and percentage of correctly classified in the prediction of PAs.

**TABLE 6:** Summary of MDD-W, MDD-IYC, and FGS-10 ≥4 characteristics relative to predicting PA of children and NPNB women^1^

	Children, *n* = 1066, PA >0.8			NPNB women, *n* = 432, PA >0.8		
	Sensitivity	Specificity	Correctly classified	Sensitivity	Specificity	Correctly classified
Iron						
FGS-10 ≥4	51	65	58	46	59	57
MDD-W (FGS-10 ≥5)	21	91	55	14	84	71
MDD-IYC (FGS-7 ≥4)	37	77	56	33	68	62
Vitamin A						
FGS-10 ≥4	66	74	71	65	69	68
MDD-W (FGS-10 ≥5)	28	95	67	31	92	72
MDD-IYC (FGS-7 ≥4)	44	79	64	43	74	64
Zinc						
FGS-10 ≥4	44	88	46	46	75	52
MDD-W (FGS-10 ≥5)	16	100	19	18	94	33
MDD-IYC (FGS-7 ≥4)	32	95	34	34	79	43
Calcium						
FGS-10 ≥4	66	59	59	63	59	59
MDD-W (FGS-10 ≥5)	25	86	81	31	85	83
MDD-IYC (FGS-7 ≥4)	50	71	69	56	69	69
Vitamin B-6						
FGS-10 ≥4	48	89	54	62	71	68
MDD-W (FGS-10 ≥5)	17	99	29	29	93	68
MDD-IYC (FGS-7 ≥4)	34	91	42	48	78	66
Vitamin B-12						
FGS-10 ≥4	85	59	60	78	59	59
MDD-W (FGS-10 ≥5)	49	87	85	56	85	85
MDD-IYC (FGS-7 ≥4)	79	72	72	78	69	69
Vitamin C						
FGS-10 ≥4	55	81	63	63	73	69
MDD-W (FGS-10 ≥5)	20	96	45	31	96	69
MDD-IYC (FGS-7 ≥4)	39	85	54	44	77	63
Folate						
FGS-10 ≥4	59	76	67	76	65	66
MDD-W (FGS-10 ≥5)	24	96	57	34	88	79
MDD-IYC (FGS-7 ≥4)	42	82	60	53	72	69
Riboflavin						
FGS-10 ≥4	57	71	64	55	61	60
MDD-W (FGS-10 ≥5)	25	95	60	24	86	74
MDD-IYC (FGS-7 ≥4)	42	80	62	39	70	64
Niacin						
FGS-10 ≥4	60	74	67	75	66	68
MDD-W (FGS-10 ≥5)	25	95	60	41	91	81
MDD-IYC (FGS-7 ≥4)	43	82	63	60	75	72
Thiamin						
FGS-10 ≥4	54	83	62	62	72	68
MDD-W (FGS-10 ≥5)	20	98	43	30	95	68
MDD-IYC (FGS-7 ≥4)	38	88	53	46	78	65

1 Values of sensitivity, specificity, and correctly classified are percentages. FGS-7, food group score based on the minimum dietary diversity for infant and young child feeding (MDD-IYC) guidelines; FGS-10, food group score based on the minimum dietary diversity for women (MDD-W) guidelines; MPA, mean probability of adequacy; NPNB, nonpregnant nonbreastfeeding; PA, probability of adequacy.

### Robustness analysis

To further test the robustness of our results, we repeated the same analysis process using the lean season data. In the lean season, diets were slightly less diverse, MPAs were lower, though energy intakes were comparable to those in the dry season (**Supplemental Table 1**). Overall, the findings on the association and performance of FGS-10 and FGS-7 were similar and confirmed the findings from the dry season (**Supplemental Tables 2** and **3**). Regression analysis also showed that the relation between FGS and MPA did not vary by season in either children or women (*P*-values >0.1 as suggested by the interaction term FGS × season) (**Supplemental Table 4**).

Robustness checks using various MPA thresholds to define micronutrient adequacy led to similar conclusions on indicators and FGS cutoffs in both children and women (**Supplemental Tables 5** and **6**).

Additional analyses were also conducted without considering a minimum threshold to count a group. These analyses showed that, without a minimum threshold, the association between MPA or PAs and FGSs became less strong. This was reflected in lower correlation coefficients in all groups and some nonsignificant correlation coefficients for iron and riboflavin in the NPNB women group (**Supplemental Table 7**). MDD-W and MDD-IYC predicted well MPA >0.75 in children and MPA >0.6 in women, but a cutoff of 5 seemed slightly more suitable for MDD-IYC because it offered a better balance between sensitivity, specificity, and percentage good classification, particularly for NPNB women (**Supplemental Table 8**).

## Discussion

This study showed that FGSs based on either the MDD-W guidelines (10 food groups, recommended for nonpregnant women) (8) or the MDD-IYC guidelines (7 food groups, recommended for 6–23-mo-old children) (7) were positively associated with the MPA and PAs of the 11 micronutrients considered in children aged 24 to 59 mo and in pregnant, breastfeeding, and NPNB women in rural Burkina Faso. In children, MDD-IYC was an adequate dichotomous indicator of “higher” MPA at the group level. MDD-W worked well to correctly identify children with low probability of micronutrient adequacy, but adapting the cutoff to 4 of 10 food groups (instead of 5 of 10) allowed for a better balance between sensitivity, specificity, and proportion of correct classification. Results for the NPNB women show the same figures for both food grouping systems.

Although FGS-10 generally performed slightly better than FGS-7 for all target groups, this study showed that both FGSs were positively correlated to MPAs in children and in pregnant, breastfeeding, and NPNB women. These results are consistent with the well-established body of evidence that dietary diversity is strongly correlated to micronutrient adequacy or density in children of various ages (5, 10–13, 35, 36) and in women of various physiological statuses (4, 9, 37) in low-income countries. We also showed that the relation between FGS and MPA did not vary by season in either children or women, which suggests that FGSs are good proxy indicators of the probability of micronutrient adequacy at all times in Burkina Faso (Supplemental Table 2). This was also the conclusion of a study in 6–12-mo-old infants in Ethiopia (36). Conversely, a recent study in 4–8-y-old children in Zambia showed that in the late rainy season, FGS-7 or FGS-10 had a flat relation with MPA, as opposed to the early rainy season and the late postharvest season when the positive association between FGS and MPA was also found (13). The authors hypothesized that this finding was mainly due to a lower food intake during the late rainy season, associated with a lower MPA and energy intake whereas FGSs remained stable. In this study, energy intakes were similar in both seasons (except for children), but MPA and FGSs were both lower in the rainy season. More in-depth analysis of dietary intake data, which is beyond the scope of the present study, is required to fully understand how seasons can affect the use and interpretation of FGSs as proxy indicators of the probability of micronutrient adequacy.

MDD-IYC (i.e., FGS-7 ≥4 food groups) was found to be a good indicator to assess micronutrient adequacy at the group level in 24–59-mo-old children. MDD-W (i.e., FGS-10 ≥5 food groups) identified correctly >90% of children with lower probability of micronutrient adequacy (MPA <0.75) but underperformed in correctly identifying the children with higher probability of micronutrient adequacy. For children, a better indicator for assessment at the group level, based on the 10 food groups recommended for MDD-W, would use a cutoff of 4 of 10 food groups to define higher probability of micronutrient adequacy. Results in NPNB women (using MPA >0.6) showed the same figures in both seasons. However, given that the cutoff of 5 of 10 food groups has been specifically validated for nonpregnant women using 9 studies from 6 countries (9), we do not recommend a change based on our study only. Indeed, our results with the cutoff of 5 of 10 food groups fit within the ranges of sensitivity and specificity found in that validation study. Specifically, 2 of 9 datasets had excellent specificity (≥90%), but sensitivity was poor (15–36%). Six of 9 datasets had higher sensitivity (57–79%) than in our study for that cutoff but were less good at correctly identifying women with lower micronutrient adequacy (specificity 55–75%). Also, the results of the robustness analysis performed on FGSs without the 10-g threshold to count as a food group favored the MDD-W cutoff in both women and children.

The optimal cutoffs in our study were also performing reasonably well at identifying individuals with low probability of covering their requirements for individual micronutrients, except for micronutrients with excellent prevalence of adequacy in the sample (PA >0.8), that is, zinc (98% in children and 90% in NPNB women) and vitamin B-6 in children (88%). Indeed, specificities for individual micronutrient PAs ranged from 58% to 98% in children, and from 58% to 95% in NPNB women. However, sensitivities (20–85%) and percentage of correctly classified (45–84%) were low.

Our results suggest that using a single standard FGS across different target groups and seasons is possible for rural children and NPNB women. The positive correlations between FGS and MPA were of the same magnitude across seasons. However, despite similar results in sensitivity and specificity for the different target groups, and across seasons and MPA cutoffs, the 2 FGSs do not predict similar MPAs in children and women. For a given FGS, MPAs for NPNB women were lower compared with those of children, which suggests that women have more inadequate micronutrient intakes (Figure 1). As such, it would be expected that different ages and physiological statuses associated with higher micronutrient requirements require different cutoffs for defining a dichotomous indicator of appropriate adequacy of the diet. In a recent study in pregnant adolescents and women in Bangladesh (whose micronutrient needs are higher than nonpregnant women), MDD-W led to a high percentage of misclassification, and the authors recommended a higher cutoff of FGS-10 ≥6 food groups (instead of ≥5) to predict MPA >0.6 in these populations (37). The optimal cutoff of ≥4 we found for FGS-10 to predict MPA >0.75 in children would be consistent with this expectation as well, because children aged 24–59 mo have lower micronutrient requirements than women (relative to their energy requirements) (38, 39). For FGS-7, we found the same optimal cutoffs for children aged 6–59 mo and NPNB women, which also corresponded to the optimal cutoff validated for children aged 6–24 mo (MDD-IYC). From an operational point of view, it would be convenient to be able to use a single FGS across populations and settings, with possibly different cutoff points to define adequate micronutrient adequacy. Our study should be replicated in other settings and target groups to assess whether MDD-IYC and/or MDD-W can be extended to other populations in both urban and rural settings and to assess what the most suitable cutoff points will be to dichotomize into low and high micronutrient adequacy for different ages and physiological statuses.

This study has a number of strengths and limitations. One particular strength is the fairly large study sample of both young children and women, at 2 seasons and across 3 regions of Burkina Faso. One limitation was the use of actual intakes (instead of usual intakes) to calculate the PA of vitamin B-12. The extreme skewness of the vitamin B-12 intake distribution due to many zero values made it impossible to generate a usual intake distribution.

Another limitation was the proportion of underreporters, which was rather high, but remained within the boundaries of other studies conducted in low- and middle-income countries (23). Moreover, our findings were not altered in robustness analyses excluding these underreporters (data not shown). Another limitation is that the FGSs used in this study were compiled using the 24-h recall data also used for MPA calculation. No standalone questionnaire was thus used to measure the food group consumption, resulting in the best-case scenario to find associations between FGS and MPA. In many settings the frequency of food group consumption is measured using simpler methods like open qualitative 24-h recalls (i.e., dietary recalls without estimating portion size) or list-based questionnaires (i.e., asking a respondent if food groups were consumed or not). Previous studies reported that the use of such simpler and more operational data collection methods to count food groups resulted in misreporting of some food groups, compared with quantitative data collection methods considered as gold standard in these studies, although their performance was deemed acceptable when operational considerations were factored in (40–42). More research is needed to inform on the most cost-effective data collection tools to compile FGSs, MDD-W, and MDD-IYC. Finally, we were unable to conduct our analysis for the sample of pregnant and breastfeeding women because we had too few observations with higher MPA. Urgent attention is needed to tackle the very low micronutrient intakes (and MPAs) found in these physiological groups; the development and validation of specific indicators with appropriate cutoff points for pregnant and breastfeeding women should be explored in populations in which more women of these physiological groups are able to better meet their dietary requirements.

Despite these limitations, we conclude that using an FGS based on either 10 or 7 food groups (as per MDD-W or the MDD-IYC guidelines) can be extended to children aged 24–59 mo to collect useful discrete proxy indicators of micronutrient adequacy of the diet across seasons in this age range in rural Sahelian contexts with similar dietary habits. The proportion of children having consumed 4 food groups (of 7 or 10 food groups) in the past 24 h can be used as a proxy indicator for assessing the probability of micronutrient adequacy in this population, regardless of the season. Multicountry validation of these indicators and standard recommendations on their use in children aged >24 mo is now needed. Special attention should also be given to pregnant and breastfeeding women in order to tackle their low micronutrient intakes.

## Supplementary Material

nxaa360_Supplemental_File

## Notes

We acknowledge the support from the Bill & Melinda Gates Foundation (grant number: OPP1149709), and the CGIAR Research Program on Agriculture for Nutrition and Health (A4NH), led by IFPRI (subgrant number A4NH-202004.040.500).

Author disclosures: The authors report no conflicts of interest.

Supplemental Figures 1 and 2 and Supplemental Tables 1–8 are available from the “Supplementary data” link in the online posting of the article and from the same link in the online table of contents at https://academic.oup.com/jn/.

Abbreviations used: EAR, estimated average requirement; FCT, food composition table; FGS, food group score; IYC, infant and young child; IYCF, infant and young child feeding; MDD, minimum dietary diversity; MDD-IYC, minimum dietary diversity for infants and young children; MDD-W, minimum dietary diversity for nonpregnant women; MPA, mean probability of adequacy; NPNB, nonpregnant nonbreastfeeding; PA, probability of adequacy; PCC, pair of caregiver and child; SELEVER, Soutenir l'Exploitation familiale pour Lancer l’Élevage des Volailles et Valoriser l’Économie Rurale.
