# Supplementary material for: Standard Minimum Dietary Diversity Indicators for Women or Infants and Young Children Are Good Predictors of Adequate Micronutrient Intakes in 24–59-Month-Old Children and Their Nonpregnant Nonbreastfeeding Mothers in Rural Burkina Faso
Source: J Nutr. 2020 Dec 16;151(2):412–22. doi: 10.1093/jn/nxaa360 (PMC7850098; doi:10.1093/jn/nxaa360)
Supplement: nxaa360_Supplemental_File [file nxaa360_supplemental_file.docx]

Standard minimum dietary diversity indicators for women or infants and young children are good predictors of adequate micronutrient intakes in 24-to-59 -month-old children and their non-pregnant non-breastfeeding mothers in rural Burkina Faso.

Diop, L.

Online Supplementary material


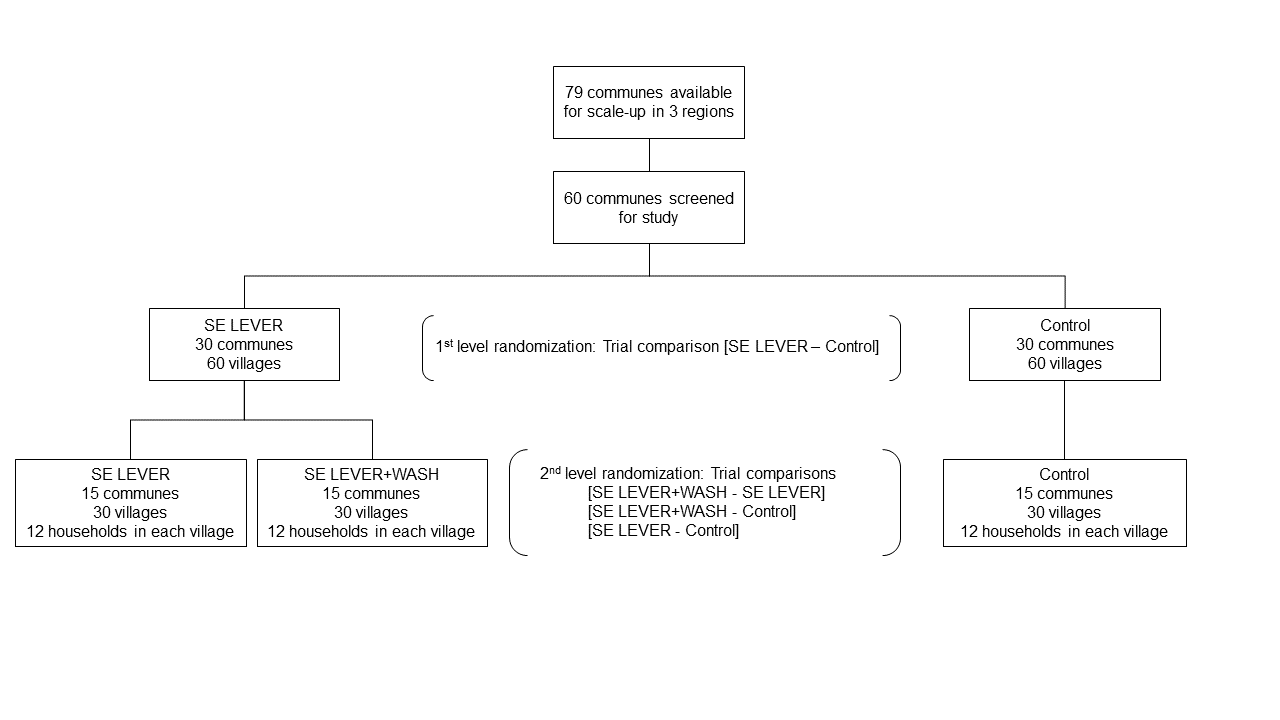
**Supplemental Figure 1:** Schematic view of the randomization

**Supplemental Figure 2:** Participants flow for the diet survey in SELEVER intervention


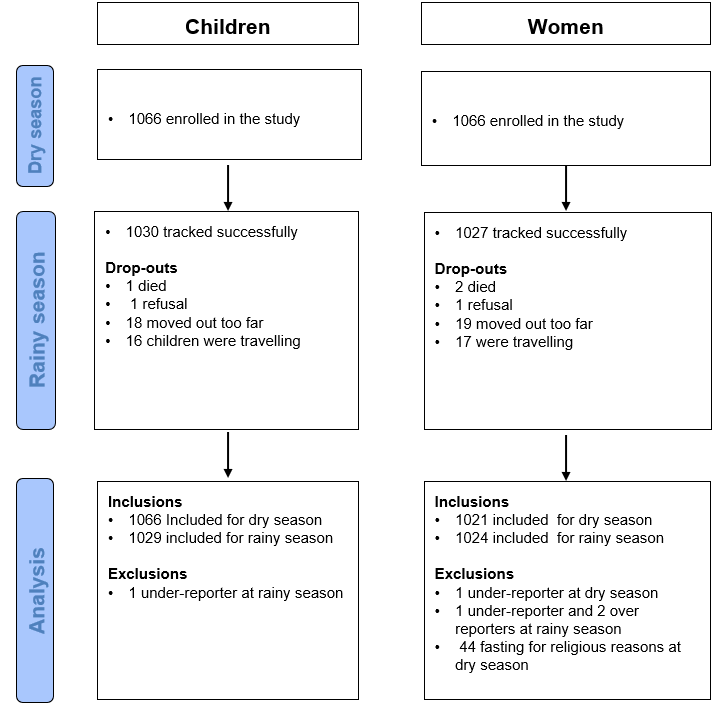


**Supplemental Table 1:** Food group scores, MPA and energy intakes for children and women, by season^1^

|  | **Children** | |  | **NPNB women** | |  | **Breastfeeding women** | |  | **Breastfeeding women** | |
| --- | --- | --- | --- | --- | --- | --- | --- | --- | --- | --- | --- |
|  | **Dry season, n = 1066** | **Lean season, n = 1029** |  | **Dry season, n = 432** | **Lean season, n = 417** |  | **Dry season, n = 403** | **Lean season, n = 345** |  | **Dry season, n = 173** | **Lean season, n = 250** |
| FGS-10 | 3.4 ± 1.2 (1-8) *** | 3.0 ± 1.0 (1-7) |  | 3.3 ± 1.2 (1-7) *** | 2.9 ± 1.0 (1-7) |  | 3.3 ± 1.2 (1-8) *** | 3.1 ± 0.97 (1-6) |  | 3.3 ± 1.0 (1-6) ** | 3.0 ± 0.92 (1-6) |
| FGS-7 | 3.1 ± 0.93 (1-6) *** | 2.9 ± 0.90 (1-5) |  | 3.0 ± 0.93 (1-5) ** | 2.9 ± 0.90 (1-5) |  | 3.1 ± 0.97(1-6) ** | 2.9 ± 0.83 (1-5) |  | 3.0 ± 0.91 (1-5) | 3.0 ± 0.87 (1-5) |
| Energy, kcal/d | 1300 ± 596 (133-4285) | 1330 ± 589 (146 - 4569) *** |  | 2064 ± 826 (230 - 5967) | 2019 ± 853 (89 - 5806) |  | 2208 ± 862 (585 - 5612) | 2210 ± 929 (474 - 5817) |  | 1930 ± 727 (403 - 4047) | 2032 ± 825 (256 - 4461) |
| MPA | 0.58 ± 0.22 (0 - 1) *** | 0.51 ± 0.22 (0 - 0.99) |  | 0.35 ± 0.23 (0 - 0.96) *** | 0.27 ± 0.21 (0 - 0.90) |  | 0.29 ± 0.2 (0 - 0.91) *** | 0.21 ± 0.2 (0 - 0.9) |  | 0.24 ± 0.19 (0 - 0.78) ** | 0.17 ± 0.14 (0 - 0.70) |

^1^Values are mean ± SD (range). Stars indicates p-values of paired t-tests comparing mean values at both seasons. (*** means p-value <0.001 and ** means p-value <0.05).

FGS-10, Food group score based on the Minimum dietary diversity for women (MDD-W) guidelines; FGS-7, Food group score based on the infant and young child feeding (MDD-IYC) guidelines; kcal/d, kilocalories per day; MPA, Mean Probability of Adequacy; NPNB, Non-Pregnant-Non-Breastfeeding.

**Supplemental Table 2:** Correlation between FGS and the MPA for children and women, by season^1^

|  | **Children** | | | |  | **NPNB women** | | | |  | **Breastfeeding women** | | | |  | **Pregnant women** | | | | |
| --- | --- | --- | --- | --- | --- | --- | --- | --- | --- | --- | --- | --- | --- | --- | --- | --- | --- | --- | --- | --- |
|  | **Dry season** | | **Lean season** | |  | **Dry season** | | **Lean season** | |  | **Dry season** | | **Lean season** | |  | **Dry season** | | **Lean season** | |  |
|  | **Without energy** | **With energy** | **Without energy** | **With energy** |  | **Without energy** | **With energy** | **Without energy** | **With energy** |  | **Without energy** | **With energy** | **Without energy** | **With energy** |  | **Without energy** | **With energy** | **Without energy** | **With**  **energy** |  |
|  | **n=1066** | | **n=1029** | |  | **n=432** | | **n=417** | |  | **n=403** | | **n=345** | |  | **n=173** | | **n=250** | |  |
| FGS-10 | 0.59*** | 0.40*** | 0.48*** | 0.39*** |  | 0.53*** | 0.31*** | 0.42*** | 0.26*** |  | 0.54*** | 0.38*** | 0.41*** | 0.26*** |  | 0.55*** | 0.39*** | 0.26** | 0.16** |  |
| FGS-7 | 0.50*** | 0.31*** | 0.47*** | 0.40*** |  | 0.42*** | 0.20*** | 0.40*** | 0.26*** |  | 0.47*** | 0.30*** | 0.37*** | 0.23*** |  | 0.52*** | 0.35*** | 0.21** | 0.13** |  |

^1^Values are Spearman’s rank correlation coefficients. Values without energy are Spearman’s rank correlation coefficient. Values with energy are Spearman’s rank partial correlation coefficients (2). Stars indicates the p-value of correlation values significance tests (*** means p-value <0.001).

FGS-10, Food group score based on the Minimum dietary diversity for women (MDD-W) guidelines; FGS-7, Food group score based on the infant and young child feeding (MDD-IYC) guidelines; MPA, Mean Probability of Adequacy; NPNB, Non-Pregnant-Non-Breastfeeding.

**Supplemental Table 3:** Summary of FGS characteristics relative to predicting MPA of children and women at lean season^1^

|  |  | **Children, n=1029, MPA>0.75** | | | |  | **NPNB women, n =417, MPA>0.6** | | | |
| --- | --- | --- | --- | --- | --- | --- | --- | --- | --- | --- |
|  |  | n | Sensitivity | Specificity | Correctly classified |  | n | Sensitivity | Specificity | Correctly classified |
| **FGS-10** | (≥ 1) | *1029* | 100 | 0.0 | 14 |  | *417* | 100 | 0.0 | 18 |
|  | (≥2) | *989* | 100 | 4.5 | 18 |  | *406* | 100 | 3.4 | 21 |
|  | (≥ 3) | *670* | 89 | 39 | 46 |  | *257* | 95 | 31 | 43 |
|  | (≥ 4) | *293* | 58 | 77 | 74 |  | *112* | 77 | 66 | 68 |
|  | **MDD-W (≥ 5)** | *67* | 18 | 95 | 84 |  | *31* | 38 | 90 | 80 |
|  | (≥ 6) | *9* | 2.7 | 99 | 86 |  | *6* | 13 | 98 | 82 |
|  | (≥ 7) | *1* | 0.7 | 100 | 86 |  | *1* | 3.8 | 100 | 82 |
|  | (> 7) | *0* | 0.0 | 100 | 86 |  | *0* | 0.0 | 100 | 82 |
|  |  |  |  |  |  |  |  |  |  |  |
| **FGS-7** | (≥ 1) | 1029 | 100 | 0.0 | 14 |  | *417* | 100 | 0.0 | 18 |
|  | (≥ 2) | 990 | 100 | 4.4 | 18 |  | *405* | 100 | 3.4 | 21 |
|  | (≥ 3) | 658 | 88 | 40 | 47 |  | *255* | 94 | 37 | 48 |
|  | **MDD-IYC (≥ 4)** | 258 | 55 | 80 | 76 |  | *97* | 57 | 74 | 71 |
|  | (≥ 5) | 32 | 11 | 98 | 86 |  | *17* | 15 | 97 | 82 |
|  | (> 5) | 0 | 0.0 | 100 | 86 |  | *0* | 0.0 | 100 | 82 |

^1^Values of sensitivity specificity and correctly classified are percentages.

FGS-10, Food group score based on the Minimum dietary diversity for women (MDD-W) guidelines; FGS-7, Food group score based on the infant and young child feeding (MDD-IYC) guidelines; MPA, Mean Probability of Adequacy; NPNB, Non-Pregnant-Non-Breastfeeding.

**Supplemental Table 4:** Results of linear regressions of FGS on MPA^1^

|  | | | | **Children ^2^, n=1063** | | **NPNB women ^3^, n=432** | |  |
| --- | --- | --- | --- | --- | --- | --- | --- | --- |
| **FGS-10** | |  | |  | |  | |  |
|  |  | | 3 groups | | 0.12*** | | 0.11*** | |
|  |  | | 4 groups | | 0.17*** | | 0.23*** | |
|  |  | | 5 groups | | 0.24*** | | 0.26*** | |
|  |  | | 6 groups or more | | 0.27*** | | 0.21*** | |
|  | **FGS-10 x lean season** | | | -0.01 | | 0.01 | |  |
| **FGS-7** |  | |  | |  | |  | |
|  |  | | 3 groups | | 0.11*** | | 0.12*** | |
|  |  | | 4 groups | | 0.16*** | | 0.13*** | |
|  |  | | 5 groups or more | | 0.23*** | | 0.23*** | |
|  | **FGS-7 x lean season** | | | 0.03 | | -0.28 | |  |

^1^Values are regression coefficients. The reference categories for FGS-10 or FGS-7 are “lower than 3 groups”. Results for pregnant women are not shown because there was an inconsistency in the coefficients obtained due to the relative weakness of the link between FGSs and MPA for this group. Results for breastfeeding women are not shown because the distribution of residuals for this model was not normal. Stars indicates the p-value of coefficient significance tests (***means p-value <0.001).

^2^ Model adjusted for sex, energy intake and whether the child is breastfed or not.

^3^ Model adjusted for age, energy intake and height.

FGS-10, Food group score based on the Minimum dietary diversity for women (MDD-W) guidelines; FGS-7, Food group score based on the infant and young child feeding (MDD-IYC) guidelines; MPA, Mean Probability of Adequacy; NPNB, Non-Pregnant-Non-Breastfeeding.

**Supplemental Table 5:** Summary of FGS characteristics relative to predicting MPA>0.8 for children^1^

|  |  | **Dry season, n = 1066** | | | |  | **Lean season, n= 1029** | | | |
| --- | --- | --- | --- | --- | --- | --- | --- | --- | --- | --- |
|  |  | n | Sensitivity | Specificity | Correctly classified |  | n | Sensitivity | Specificity | Correctly classified |
| **FGS-10** | (≥ 1) | *1066* | 100 | 0.0 | 19 |  | *1029* | 100 | 0.0 | 9.2 |
|  | (≥ 2) | *1033* | 100 | 3.8 | 23 |  | *989* | 100 | 4.3 | 13 |
|  | (≥ 3) | *811* | 97 | 29 | 42 |  | *670* | 91 | 37 | 42 |
|  | (≥ 4) | *458* | 75 | 65 | 67 |  | *293* | 63 | 75 | 74 |
|  | **MDD-W (≥ 5)** | *160* | 40 | 91 | 81 |  | *67* | 21 | 95 | 88 |
|  | (≥ 6) | *43* | 13 | 98 | 81 |  | *9* | 4.2 | 99 | 91 |
|  | (≥ 7) | *7* | 2.9 | 100 | 81 |  | *1* | 1.1 | 100 | 91 |
|  | (≥ 8) | *1* | 0.48 | 100 | 81 |  | *0* | 0.0 | 100 | 91 |
|  | (> 8) | *0* | 0.0 | 100 | 81 |  | - | - | - | - |
|  |  |  |  |  |  |  |  |  |  |  |
| **FGS-7** | (≥ 1) | 1066 | 100 | 0.0 | 19 |  | 1029 | 100 | 0.0 | 9.2 |
|  | (≥ 2) | 1034 | 100 | 3.7 | 22 |  | 990 | 100 | 4.2 | 13 |
|  | (≥ 3) | 759 | 93 | 34 | 45 |  | 658 | 89 | 39 | 43 |
|  | **MDD-IYC (≥4)** | 327 | 59 | 76 | 73 |  | 258 | 61 | 79 | 77 |
|  | (≥ 5) | 59 | 18 | 97 | 82 |  | 32 | 15 | 98 | 90 |
|  | (≥ 6) | 4 | 0.97 | 100 | 81 |  | 0 | 0.0 | 100 | 91 |
|  | (> 6) | 0 | 0.0 | 100 | 81 |  | - | - | - | - |

^1^Values of sensitivity specificity and correctly classified are percentages.

FGS-10, Food group score based on the Minimum dietary diversity for women (MDD-W) guidelines; FGS-7, Food group score based on the infant and young child feeding (MDD-IYC) guidelines; MPA, Mean Probability of Adequacy.

**Supplemental Table 6:** Summary of FGS characteristics relative to predicting MPA>0.7 for NPNB women^1^

|  |  | **Dry season, n =432** | | | |  | **Lean season, n = 417** | | | |
| --- | --- | --- | --- | --- | --- | --- | --- | --- | --- | --- |
|  |  | n | Sensitivity | Specificity | Correctly classified |  | n | Sensitivity | Specificity | Correctly classified |
| **FGS-10** | (≥ 1) | *432* | 100 | 0.0 | 11 |  | *417* | 100 | 0.0 | 4.3 |
|  | (≥ 2) | *420* | 100 | 3.1 | 14 |  | *406* | 100 | 2.8 | 7.0 |
|  | (≥ 3) | *319* | 94 | 29 | 36 |  | *257* | 89 | 40 | 42 |
|  | (≥ 4) | *181* | 81 | 63 | 65 |  | *112* | 61 | 75 | 74 |
|  | **MDD-W (≥ 5)** | *67* | 45 | 88 | 83 |  | *31* | 39 | 94 | 92 |
|  | (≥ 6) | *17* | 13 | 97 | 88 |  | *6* | 11 | 99 | 95 |
|  | (≥ 7) | *3* | 6.4 | 100 | 90 |  | *1* | 5.6 | 100 | 96 |
|  | (> 7) | *0* | 0.0 | 100 | 89 |  | *0* | 0.0 | 100 | 96 |
|  |  |  |  |  |  |  |  |  |  |  |
| **FGS-7** | (≥ 1) | *432* | 100 | 0.0 | 11 |  | *417* | 100 | 0.0 | 4.3 |
|  | (≥2) | *420* | 100 | 3.1 | 14 |  | *405* | 100 | 3.0 | 7.2 |
|  | (≥ 3) | *295* | 94 | 35 | 41 |  | *255* | 89 | 40 | 42 |
|  | **MDD-IYC (≥ 4)** | *138* | 64 | 72 | 71 |  | *97* | 56 | 78 | 77 |
|  | (≥ 5) | *22* | 15 | 96 | 87 |  | *17* | 39 | 98 | 95 |
|  | (> 5) | 0 | 0.0 | 100 | 89 |  | 0 | 0.0 | 100 | 96 |

^1^Values of sensitivity specificity and correctly classified are percentages. FGS-10, Food group score based on the Minimum dietary diversity for women (MDD-W) guidelines; FGS-7, Food group score based on the infant and young child feeding (MDD-IYC) guidelines; MPA, Mean Probability of Adequacy; NPNB, Non-Pregnant-Non-Breastfeeding.

**Supplemental Table 7:** Correlation between FGS and PAs of 11 micronutrients of MPA for children and women without considering a minimum threshold to count a group^1^

|  | Children (n=1066) | | NPNB women (n=432) | | Breastfeeding women (n=403) | | Pregnant women (n=173) | |
| --- | --- | --- | --- | --- | --- | --- | --- | --- |
|  | FGS-10 | FGS-7 | FGS-10 | FGS-7 | FGS-10 | FGS-7 | FGS-10 | FGS-7 |
| MPA | 0.44*** | 0.34*** | 0.41*** | 0.25*** | 0.36*** | 0.26*** | 0.40*** | 0.37*** |
| PA iron | 0.12*** | 0.11*** | 0.04 | 0.02 | 0.14*** | 0.15*** | 0.13*** | 0.18** |
| Vitamin A | 0.45*** | 0.30*** | 0.44*** | 0.28*** | 0.36*** | 0.23*** | 0.37*** | 0.27*** |
| Zinc | 0.13*** | 0.10*** | 0.21*** | 0.14*** | 0.22*** | 0.17*** | 0.27*** | 0.31*** |
| Calcium | 0.13*** | 0.11*** | 0.20*** | 0.16*** | 0.11** | 0.1** | 0.18** | 0.17** |
| Vitamin B6 | 0.25*** | 0.19*** | 0.30*** | 0.19*** | 0.19*** | 0.15*** | 0.18** | 0.21*** |
| Vitamin B12 | 0.30*** | 0.39*** | 0.27*** | 0.39*** | 0.29*** | 0.34*** | 0.33*** | 0.37*** |
| Vitamin C | 0.38*** | 0.27*** | 0.45*** | 0.27*** | 0.30*** | 0.18*** | 0.37*** | 0.22*** |
| Folate | 0.32*** | 0.19*** | 0.34*** | 0.18*** | 0.24*** | 0.12** | 0.19*** | 0.14* |
| Riboflavin | 0.27*** | 0.22*** | 0.15*** | 0.08 | 0.13*** | 0.14*** | 0.24** | 0.27*** |
| Niacin | 0.30*** | 0.25*** | 0.26*** | 0.18*** | 0.25*** | 0.22*** | 0.36*** | 0.38*** |
| Thiamin | 0.38*** | 0.26*** | 0.44*** | 0.25*** | 0.36*** | 0.22*** | 0.43*** | 0.33*** |

^1^Values are Spearman’s rank correlation coefficients. Stars indicates the p-value of correlation values significance tests (*** means p-value <0.001 and **means p-value <0.05).

FGS-10, Food group score based on the Minimum dietary diversity for women (MDD-W) guidelines; FGS-7, Food group score based on the infant and young child feeding (MDD-IYC) guidelines; MPA, Mean Probability of Adequacy; NPNB, Non-Pregnant-Non-Breastfeeding; PA, Probability of Adequacy.

**Supplemental Table 8:** Summary of FGS characteristics relative to predicting MPA of children and NPNB women without considering a minimum threshold to count a group^1^

|  |  | Children, n=1066, MPA>0.75 | | | |  | NPNB women, n =432, MPA>0.6 | | | |
| --- | --- | --- | --- | --- | --- | --- | --- | --- | --- | --- |
|  |  | n | Sensitivity | Specificity | Correctly classified |  | n | Sensitivity | Specificity | Correctly classified |
| FGS-10 | (≥ 1) | *1066* | 100 | 0.0 | 30 |  | *432* | 100 | 0.0 | 19 |
|  | (≥ 2) | *1064* | 100 | 0.3 | 30 |  | *430* | 100 | 0.60 | 20 |
|  | (≥ 3) | *985* | 98 | 10 | 36 |  | *393* | 98 | 11 | 27 |
|  | (≥4) | *715* | 86 | 41 | 55 |  | *280* | 86 | 40 | 49 |
|  | **MDD-W (≥ 5)** | *361* | 60 | 77 | 72 |  | *140* | 60 | 74 | 72 |
|  | (≥ 6) | *127* | 23 | 93 | 72 |  | *45* | 23 | 93 | 79 |
|  | (≥ 7) | *32* | 6.6 | 99 | 71 |  | *10* | 6.0 | 99 | 81 |
|  | (≥ 8) | *3* | 0.3 | 100 | 70 |  | *1* | 0.0 | 100 | 81 |
|  | (> 8) | *0* | 0.0 | 100 | 70 |  | *0* | 0.0 | 100 | 81 |
|  |  |  |  |  |  |  |  |  |  |  |
| FGS-7 | (≥ 1) | 1066 | 100 | 0.0 | 30 |  | *432* | 100 | 0.0 | 19 |
|  | (≥ 2) | 1064 | 100 | 0.3 | 30 |  | *430* | 100 | 0.60 | 20 |
|  | (≥ 3) | 974 | 97 | 11 | 37 |  | *385* | 96 | 13 | 29 |
|  | **MDD-IYC (≥ 4)** | 576 | 73 | 54 | 60 |  | *237* | 76 | 50 | 55 |
|  | (≥ 5) | 155 | 26 | 90 | 71 |  | *64* | 23 | 87 | 75 |
|  | (≥ 6) | 11 | 2.2 | 99 | 70 |  | *2* | 0.0 | 99 | 80 |
|  | (> 6) | 0 | 0.0 | 100 | 70 |  | 0 | 0.0 | 100 | 80 |

^1^Values of sensitivity specificity and correctly classified are percentages.

FGS-10, food group score based on the Minimum dietary diversity for women (MDD-W) guidelines; FGS-7, Food group score based on the infant and young child feeding (MDD-IYC) guidelines; MPA, Mean Probability of Adequacy; NPNB, Non-Pregnant-Non-Breastfeeding.
